# Acetic acid-assisted controlled hydrolysis for Br-content-dependent emission color tuning in mixed-halide BiOCl_1−*x*_Br_*x*_ systems

**DOI:** 10.1039/d6na00183a

**Published:** 2026-10-27

**Authors:** Afsah Saleem, Asad M. Khan, Ahmad K. Badawi, Bushra Ismail

**Affiliations:** a Department of Chemistry, COMSATS University Islamabad Abbottabad Campus-22060 Pakistan bushraismail@cuiatd.edu.pk afsahsaleem110@gmail.com amkhan@cuiatd.edu.pk +92 992 383595 +92 992 383592; b Civil Engineering Department, El-Madina Higher Institute for Engineering and Technology Giza 12588 Egypt dr.ahmedkaram91@gmail.com

## Abstract

Controlling the emission color of bismuth oxyhalides (BiOX) remains a significant challenge, mainly because current strategies rely on multiple reagents, heterostructures, or energy-intensive synthetic routes. These approaches often disrupt the inherent structure–property relationship in these materials. In the current study, mixed bismuth oxyhalides (*x* = 0.0–1.0) were prepared using a simple acetate-assisted coprecipitation process. Glacial acetic acid regulated the release of Bi^3+^ ions while stabilizing the solution pH, which allowed controlled hydrolysis and halide incorporation. This strategy facilitated the preparation of BiOCl_1−*x*_Br_*x*_ mixed-halides across the investigated compositional range (*x* = 0.0–1.0). All samples retained a tetragonal matlockite structure, matched with ICDD card No. 00-006-0249 (BiOCl) and 00-009-0393 (BiOBr), as confirmed by X-ray diffraction (XRD) analysis. As the Br^−^ content increased, the diffraction peaks shifted towards lower 2*θ* values. This is due to the replacement of smaller Cl^−^ ions (ionic radius = 1.81 Å) with larger Br^−^ ions (ionic radius = 1.96 Å), resulting in the gradual lattice expansion. To this effect, the lattice parameters increased from *a* = 3.90 Å and *c* = 7.41 Å (*x* = 0.0) to *a* = 3.97 Å and *c* = 8.31 Å (*x* = 1.0), indicating successful compositional modulation. It was observed that the Cl-rich sample (*x* = 0.0) exhibited thin flake-like morphology under scanning electron microscopy (SEM), whereas the Br-rich sample (*x* = 1.0) had more aggregated microplates. UV-vis spectroscopy revealed systematic tuning of the electronic structure, showing a gradual redshift of the absorption edge as the Br^−^ content increased. The band gap decreased from 2.70 eV to 2.18 eV, due to a stronger contribution of Br^−^ 4p orbitals to the valence band maximum. The formation of shoulders and broad absorption further explained the presence of Bi–Cl and Bi–Br bonding environments. The photoluminescence response was directly affected by these electronic changes. The emissions, including near-band-edge (∼330 nm), shallow, and deep defect emissions (∼420–480 nm), created a smooth color shift from blue to green-yellow. CIE chromaticity coordinates affirmed tunability of color with color purity exceeding 80 ± 5%. Based on optical analysis, a schematic band diagram shows an increase in the valence band with Br^−^ content. Overall, halide substitution in BiOCl_1−*x*_Br_*x*_ controls lattice expansion, modulates band gap, and tunes emission color. The acetate-assisted hydrolysis methodology is thus a scalable platform for luminescent BiOCl_1−*x*_Br_*x*_ materials without the use of surfactants or any external pH-regulator.

## Abstract 2

Controlling the emission color of bismuth oxyhalides (BiOX) remains a significant challenge, mainly because current strategies rely on multiple reagents, heterostructures, or energy-intensive synthetic routes.

## Graphical abstract

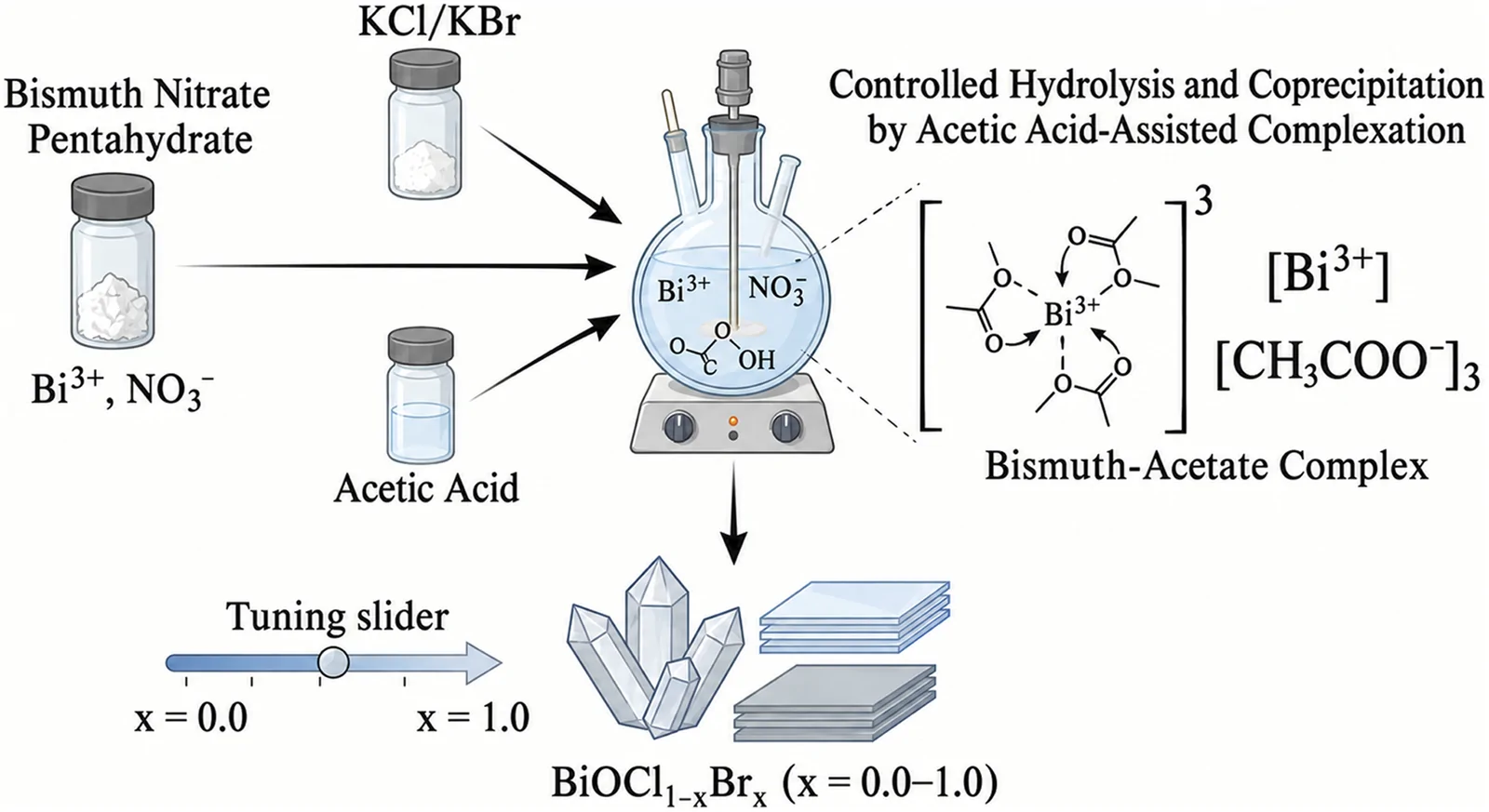

## 1. Introduction

Bismuth oxyhalides (BiOX, X = Cl, Br) have a layered tetragonal matlockite-type (PbFCl-type) structure.^1^ They consist of slabs of positively charged [–Bi–O–Bi–O–]^2+^ alternately stacked with halide (X^−^) layers along the *c*-axis, as shown in Fig. 1. This arrangement produces an anisotropic structure with stronger in-plane and weaker out-of-plane bonding.^2^ This generates internal electric fields, which enhance the spatial separation of photogenerated charge carriers and inhibit electron–hole recombination.^3^ This structural feature, therefore, increases optical and electronic performance. The electronic structure of BiOX is based on the hybridization of Bi 6s, O 2p, and halide p orbitals. Although BiOCl is highly crystalline and chemically stable, its wide band gap restricts the emission to the ultraviolet range.^4^ In contrast, BiOBr has a narrower band gap, which enables visible emission.^5^ However, neither BiOCl nor BiOBr itself offers wide-range visible-spectrum tunability.^6^ Partial substitution of Br^−^ in BiOCl offers a good pathway to actively tune lattice parameters, defect chemistry, and band structure, which control visible-light emission.^7^

**Fig. 1:**
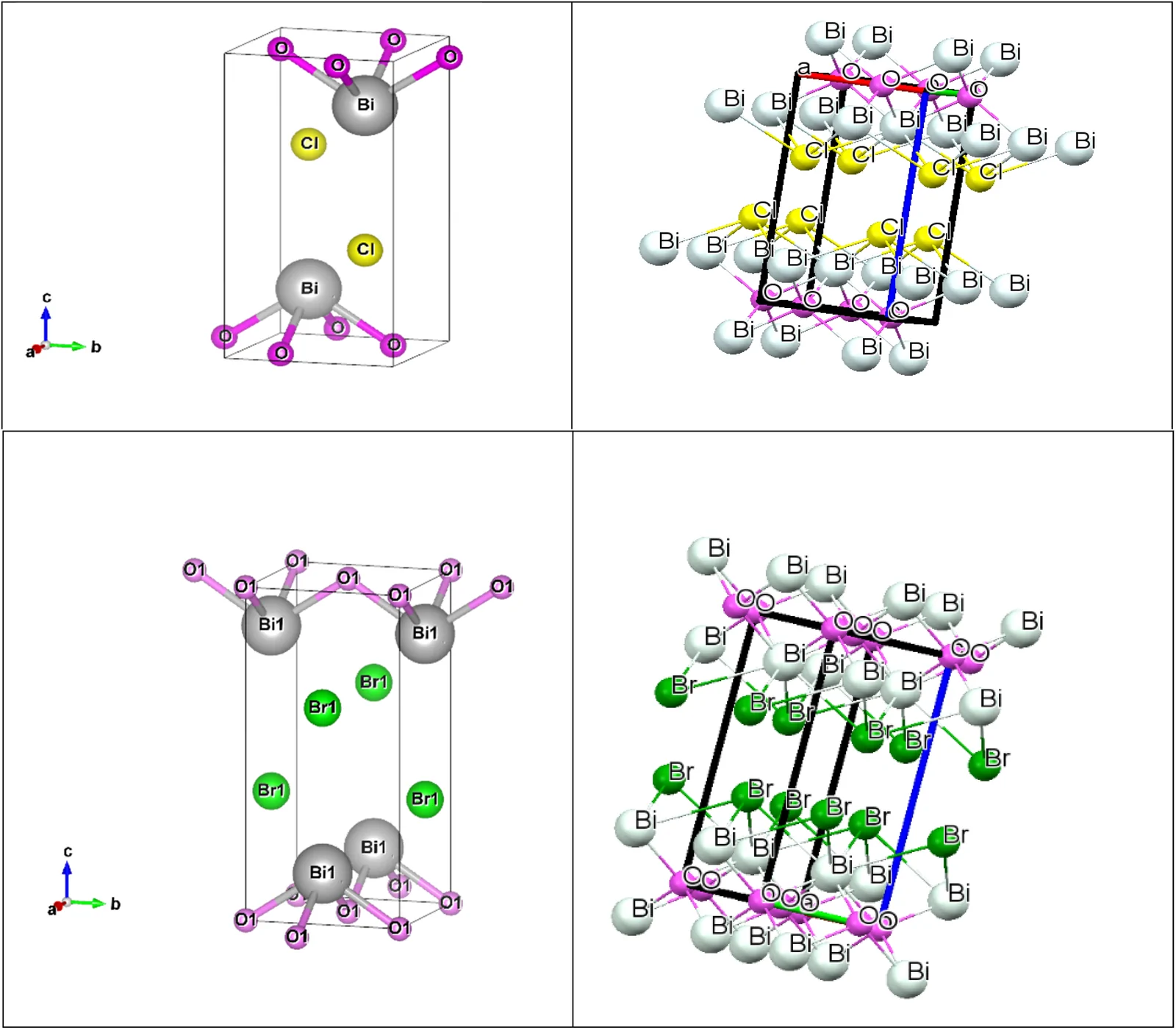
Layered matlockite-type tetragonal crystal structure of BiOX (X = Cl, Br) showing alternating [Bi–O–Bi–O]^2+^ slabs and halide layers along the *c*-axis.

A thorough literature survey of the mixed halide BiOCl_1−*x*_Br_*x*_ reveals several challenges, including synthetic complexity, limited compositional structure–property tunability, and restricted application focus, as outlined in Table 1.

**Table 1:** Reported research gaps in mixed-halide BiOCl_1−*x*_Br_*x*_ systems based on literature analysis

Category	Limitation/gap	References
Synthetic complexity	Requires multiple reagents	8
	Energy-intensive synthesis	2
Compositional and structure–property tunability	Altered intrinsic properties due to dopants or heterostructures	9
	Limited compositional range explored	10
	Incomplete bandgap–emission mapping	1
Limited application focus	Mostly restricted to photocatalysis	8

Most reported synthesis routes for BiOX involve multiple chemical reagents, such as surfactants, mineralizers, or extrinsic pH modulators.^2^ These routes increase synthetic complexity and often lead to uncontrolled nucleation and impurities. Consequently, hydrothermal and solvothermal techniques dominate, despite being energy-intensive, time-consuming, and difficult to scale.^3,11–14^

In the BiOX, structure–property tuning is often done through the use of dopants or heterostructures.^15,16^ Nevertheless, these strategies can tend to disrupt the inherent structural, electronic, and optical properties of BiOX, and hence the underlying structure–property relationship.^9^ Similar limitations and property modulation strategies have also been reported in related multifunctional semiconductor systems, where defect control and compositional tuning play a central role.^17–19^ The coprecipitation has not been studied fully previously, mainly because of the rapid, uncontrolled hydrolysis of Bi^3+^ in aqueous solution, which leads to inadequate incorporation of halides and morphological asymmetry.^3^ Furthermore, studies of BiOCl_1−*x*_Br_*x*_ have largely been limited to few composition ranges (*x* = 0.25 or 0.5).^10^ Photocatalytic performance is highlighted in most previous work, with luminescence and optical emission still under-explored despite their application in photonic and optoelectronic applications.^1,20^ The present acetate-assisted hydrolysis route allows for a single-source controlled release of Bi^3+^ ions instead of conventional hydrothermal and solvothermal methods, which involve multiple mineralizers, surfactants and high-pressure conditions, resulting in limited compositional control and may lead impurity formation. It leads to progressive incorporation of the halides over the investigated compositional range (*x* = 0.0–1.0) at mild conditions and inhibits uncontrolled nucleation and secondary phase development. Therefore, it offers higher phase purity, defect regulation, and compositional tunability than the previously reported BiOX synthesis methods. The importance of compositional control and defect modulation in tailoring optical and electronic properties has also been well demonstrated in recent advances of multifunctional semiconductor based and chalcopyrite based system.^5,21–23^ It should be noted that the hybridization of Bi 6s, O 2p, and halide p orbitals determines the electronic structure of BiOX, which provides the basis of systematic tuning of optical activity in the visible range (blue, green, and yellow).^24,25^

To address these drawbacks, a simple acetate-assisted coprecipitation method was used in this work. The glacial acetic acid was utilized for pH-regulator and complexing agent. To systematically study the lattice evolution, electronic structure and emission behavior, BiOCl_1−*x*_Br_*x*_ powders were synthesized over a wide compositional range (*x* = 0.0, 0.3, 0.5, 0.7, 1.0).

The results indicate a structure–property relationship between halide composition, band-gap tuning, and defect-assisted visible-light emission. Homogeneous nanosheets and microplates were produced using coprecipitation method. This work explores the luminescence and visible light emission properties of bismuth oxyhalide material, emphasizing its potential applications in luminescent devices. The study suggests the halide-driven emission tuning for heterostructures with a low-temperature acetate-assisted hydrolysis, which is not possible in other BiOCl_1−*x*_Br_*x*_ studies where multiple reagents or heterostructures or complex synthetic routes are used.

## 2. Experimental

### 2.1. Synthesis

All chemicals were of analytical grade and were used without further purification. BiOCl_1−*x*_Br_*x*_ (*x* = 0.0, 0.3, 0.5, 0.7, and 1.0) solids were synthesized *via* a controlled hydrolysis coprecipitation route.^10^ In a typical procedure, 24.25 g of bismuth nitrate pentahydrate [Bi(NO_3_)_3_·5H_2_O] was added to 200 mL of 4 M aqueous acetic acid prepared from glacial acetic acid and magnetically stirred at 80 °C to obtain a stable homogeneous precursor solution.

Glacial acetic acid acted as both a Bi^3+^ complexing agent and a pH-modulating medium, thus inhibiting rapid hydrolysis. The acetate formed a strong bismuth-acetate coordination complex that prevented uncontrolled hydrolysis, which is common in aqueous solution. In the absence of acetate, Bi^3+^ hydrolysis may lead to undesired side reactions (*e.g.*, the formation of bismuth hydroxide or basic bismuth nitrate species) such as:

1 Bi^3+^ + 3OH^−^ → Bi(OH)_3_↓

2 Bi^3+^ + NO^−^_3_ + 2OH^−^ → BiONO_3_↓ + H_2_O

3 2Bi(OH)_3_ → Bi_2_O_3_ + 3H_2_O

These reactions may produce irregular morphologies, halide segregation, and secondary phases, which explains why conventional coprecipitation is rarely applied in BiOX studies.

In the acetate-rich medium, Bi^3+^ formed stable bismuth-acetate complexes:

4 Bi^3+^ + 3CH_3_OO^−^ → [Bi(CH_3_COO)_3_]

This complexation moderated the concentration of free Bi^3+^ ions, buffered the solution, and enabled their gradual release. The acetate-assisted regulation of Bi^3+^ release was particularly important for maintaining controlled nucleation and phase evolution in the mixed-halide system. By slowing hydrolysis, the acetate medium minimized local supersaturation and reduced the probability of impurity phase formation or halide-segregated regions during precipitation. This controlled reaction environment facilitated homogeneous incorporation of Cl^−^ and Br^−^ ions into the BiOX lattice, particularly in Br-rich compositions where rapid precipitation is normally favored due to lower solubility. Consequently, the acetate-assisted approach improved compositional uniformity and stabilized the tetragonal BiOCl_1−*x*_Br_*x*_ phase throughout the investigated compositional range. When halide ions were added dropwise, the reaction proceeded preferentially toward the desired BiOCl_1−*x*_Br_*x*_ phase:

5 Bi^3+^ + (1−*x*)Cl^−^ + *x*Br^−^ + 2OH^−^ → BiOCl_1−*x*_Br_*x*_↓ + H_2_O

This regulated hydrolysis enabled a balance between the release of Bi^3+^ and halide supply, thus reducing side reactions and halide segregation. This is highly pronounced in Br-rich compositions, where the precipitation of BiOX is rapid due to its low solubility. Stoichiometric amounts of KCl and KBr were dissolved in 40 mL of deionized water to obtain the desired Cl^−^/Br^−^ ratios (10 : 0, 7 : 3, 5 : 5, 3 : 7, 0 : 10). The bismuth-acetate precursor was then added in halide solution dropwise with constant stirring at 80 °C. The precipitates were subsequently washed using ethanol and deionized water. The obtained products were dried in an oven. Calcination at 250 °C was performed at a heating rate of 5 °C min^−1^ for 2 h, which enhanced crystallinity without forming Bi_2_O_3_. The protocol has other advantages. It does not require any extra surfactants or several pH regulators that are used in previous techniques. This method allowed the additions to be controlled, the defects to be regulated, and predictable morphology characteristics to be attained, which were otherwise not achievable in previous BiOX synthesis.

The proposed schematic pathway for the acetate-controlled hydrolysis of Bi^3+^ and precipitation of the BiOCl_1−*x*_Br_*x*_ phase is shown in Fig. 2. The scheme suggests the proposed role of acetate coordination in the modulation of the hydrolysis of Bi^3+^ and the interaction of Bi-containing species with Cl^−^/Br^−^ in the formation of BiOX. The proposed pathway is in good agreement with the hydrolysis pathway of Bi^3+^ and the reported synthesis procedure of bismuth oxyhalides.^26^ The scheme, however, does not represent the individual intermediate species or steps in the reaction mechanism, but it is presented as a possible pathway for the formation.

**Fig. 2:**
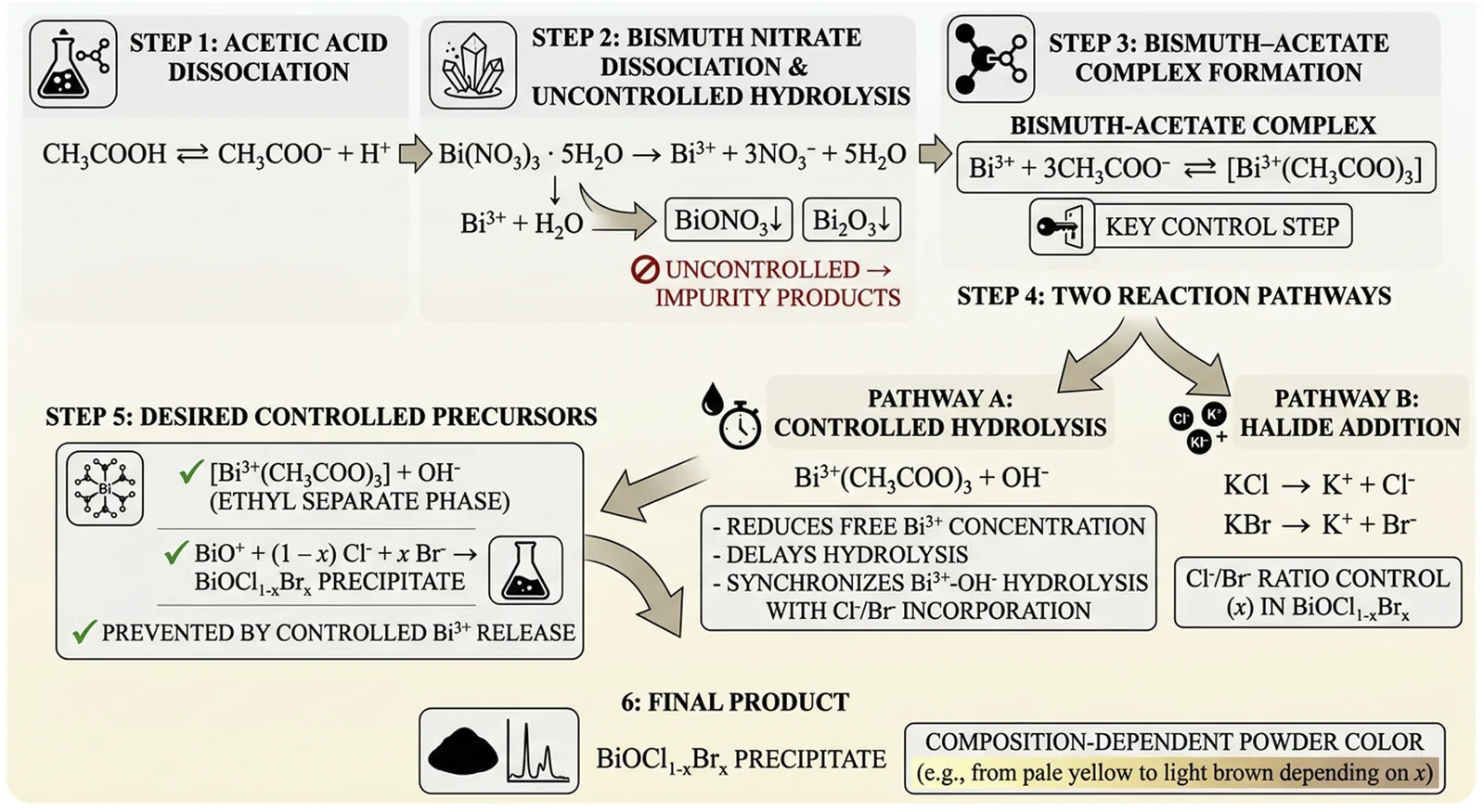
Proposed schematic formation pathway of BiOCl_1−*x*_Br_*x*_ through acetate-controlled hydrolysis and controlled halide precipitation.

### 2.2. Characterization

The crystallinity and phase purity of synthesized BiOCl_1−*x*_Br_*x*_ samples were evaluated using X-ray diffraction (XRD). Diffraction patterns were recorded using a Shimadzu XRD-7000 diffractometer with Cu Kα radiation (*λ* = 1.5406 Å) operated at 40 kV. The morphology was examined using a scanning electron microscope (SEM). Morphological observations were conducted using a JSM-5910 microscope (JEOL, Japan). UV-vis absorption spectra were recorded using a UV-vis spectrophotometer. Measurements were carried out using a specificord 200+ instrument (Analytik Jena, Germany). The BiOCl_1−*x*_Br_*x*_ samples were analysed using a photoluminescence (PL) spectrometer equipped with a 150 W xenon (Xe) lamp as an excitation source.

## 3. Results and discussion

X-ray diffraction (XRD) was used to determine the phase composition and lattice structure of the synthesized powders.^27^ The results confirmed that BiOCl_1−*x*_Br_*x*_ (*x* = 0.0–1.0) powders prepared *via* acetate-assisted hydrolysis formed a tetragonal matlockite structure throughout the investigated compositional range. No secondary phases (*e.g.*, BiONO_3_ or Bi_2_O_3_) were observed, as shown in the XRD patterns in Fig. 3. The diffraction patterns show a good indexing to the standard ICDD patterns of BiOCl (00-006-0249) and BiOBr (00-009-0393) as shown in Fig. 3. No secondary phases (*e.g.*, BiONO_3_ or Bi_2_O_3_) were observed, as shown in the XRD patterns. The diffraction peaks observed were well matched with the tetragonal matlockite-type structure of BiOCl and BiOBr, indicating the formation of BiOCl_1−*x*_Br_*x*_ system. These results agreed with the earlier reports on BiOCl_1−*x*_Br_*x*_^15^_,_ which confirmed the formation of mixed-halide BiOCl_1−*x*_Br_*x*_ (*x* = 0.0–1.0) solids under low-temperature synthesis conditions.

**Fig. 3:**
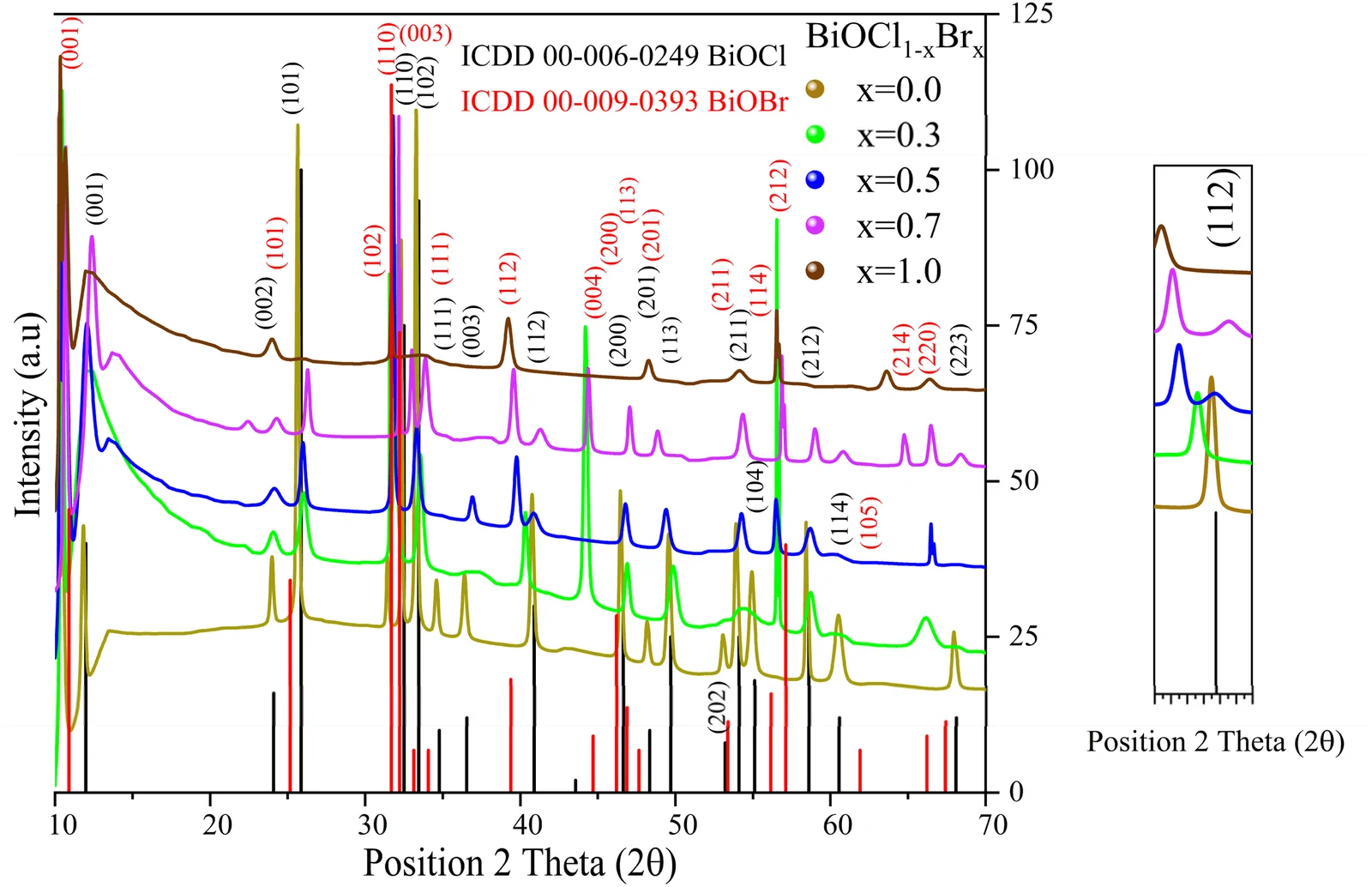
XRD patterns of BiOCl_1−*x*_Br_*x*_ (*x* = 0.0, 0.3, 0.5, 0.7, 1.0) synthesized by the acetate-assisted hydrolysis co-precipitation method.

The typical diffraction peaks, including the (002) and the (112) planes, shifted towards lower 2*θ* values as Br^−^ content increased. This systematic shift is due to the expansion of the lattice by replacing smaller Cl^−^ ions with larger Br^−^ ions. Peak symmetry and phase purity were maintained in all compositions, without detectable secondary phases.

The lattice parameters (*a* and *c*) of the tetragonal crystal structure were determined using the interplanar spacing between the diffraction angle and the miller indices of the corresponding crystallographic planes using the following equation:6
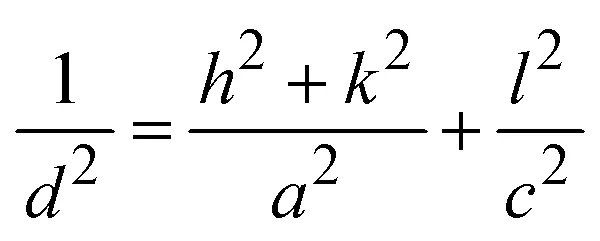


The crystallographic parameters (*a* and *c*) were calculated from the indexed XRD peaks using the interplanar spacing relations for the tetragonal crystal system. In this system, specific diffraction planes contribute selectivity to individual lattice parameters depending on the miller indices. For example, for the (001) peak, “*h* = 0” and “*k* = 0”, therefore the *d*-spacing equation depends only on “*c*”. Similarly, for the (110) peak, “*l* = 0” and the *d*-spacing depends primarily on the lattice parameter “*a*”. By using such symmetry-selected peaks, independent values of “*a*” and “*c*” were obtained from different diffraction peaks. The final lattice parameters were then determined by combining the results from multiple peaks to ensure consistency and reduced experimental error. Table 2 summarizes the lattice parameters (*a* and *c*) of BiOCl_1−*x*_Br_*x*_ powders. There was no substantial change in a parameter at 3.90 Å as the concentration of Br^−^ increased from *x* = 0.0 to 0.7. However, the “*a*” parameter slightly increased to 3.97 Å at *x* = 1.0, while the “*c*” parameter gradually increased from 7.36 Å to 8.31 Å with increasing of the Br^−^ concentration. These lattice parameters are approximated to an error of less than ±0.03Å, depending on the instrumental resolution of the XRD apparatus. This incremental change in lattice parameters is likely to occur due to the progressive substitution of smaller Cl^−^ ions (ionic radius = 1.81 Å) by larger Br^−^ ions (ionic radius = 1.96 Å) and implies a predictable composition-dependent lattice expansion.^28^

**Table 2:** Lattice parameters, crystallite size, and 2*θ* values of BiOCl_1−*x*_Br_*x*_ (*x* = 0.0, 0.3, 0.5, 0.7, 1.0)

BiOCl_1−*x*_Br_*x*_	Lattice parameters		Crystal size (nm)	Cell volume V (Å^3^)	2*θ* values of peaks crystal plane	
	a(Å) ± 0.03 Å	c(Å) ± 0.03 Å			(002)	(112)
ICDD 06-0249	3.89	7.36	—	111.57	24.0	40.8
*x* = 0.0	3.90	7.41	46	112.70	24.0	40.8
*x* = 0.3	3.90	7.41	46	112.71	24.0	40.6
*x* = 0.5	3.90	7.45	47	113.32	24.0	39.7
*x* = 0.7	3.90	7.46	48	113.47	24.0	39.6
*x* = 1.0	3.97	8.31	50	130.97	23.9	39.3

The XRD patterns indicate the tetragonal matlockite-type structure across the compositional range of BiOCl_1−*x*_Br_*x*_, with the peaks observed matching those of the BiOCl and BiOBr reference patterns. The progressive shift of peaks toward lower 2*θ* values and the progressive increase of lattice parameters suggest composition-dependent lattice expansion, which is indicative of progressive incorporation of Br^−^. No distinct peaks attributable to Bi_2_O_3_ or BiONO_3_ were detected within the instrumental resolution. The Scherrer equation was also used to estimate the crystallite sizes:7
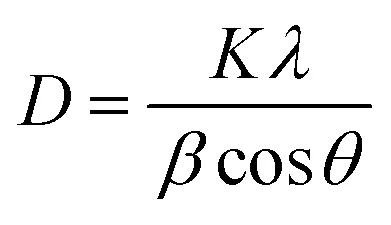
where *K* is the shape factor, *λ* is the wavelength of Cu Kα radiation (*λ* = 1.5405 Å), *β* is the diffraction peak broadening, and *θ* is the Bragg angle.^29^ In the present study, a shape factor of “*k* = 0.89” was used, which is typical of a quasi-two-dimensional nanocrystalline morphology. It is assumed that these are the structural characteristics of layered BiOCl_1−*x*_Br_*x*_ structures, and this is also supported by the SEM analysis, which shows the presence of nanosheet and plate-like structures. The anisotropic layered growth of the crystallite means that the “*K*” value chosen is a good approximation for the crystallite size estimation. The calculated crystalline size gradually increased from 46 nm for BiOCl_1−*x*_Br_*x*_ (*x* = 0.0) to approximately 50 nm in Br-rich powders. The incorporation of the bromide causes the crystal growth to increase which can be attributed to weak Bi–Br interactions that increase the mobility of the atoms in the lattice and is responsible for the lattice relaxation. Systematic weakening of Bi–X bond and structural relaxation in the layered BiOX framework were induced by the lattice expansion caused by progressive substitution of Cl^−^ by larger Br^−^ ions. This relaxation may promote the creation of halogen vacancies (VCl/VBr), which could cause local lattice distortions that create defect-rich environments in [Bi_2_O_2_]^2+^–X^−^ layers. These defects and associated structural perturbations may alter the local density of states at the band edges and could create localized energy levels and band-tail states. Hence, the overall combination of defect generation with orbital interaction (Bi 6s–O 2p–Br 4p) may contribute to radiative recombination pathways and structurally explains the tunable photoluminescence of BiOCl_1−*x*_Br_*x*_.

The compositional parameter (*x* = 0.0–1.0) in BiOCl_1−*x*_Br_*x*_ was systematically varied by precisely adjusting the ratio of Cl^−^ precursor to Br^−^ precursor during the synthesis. The formation of BiOCl_1−*x*_Br_*x*_ was confirmed by X-ray diffraction (XRD), which showed systematic peak shifts without the appearance of secondary phases, indicating successful lattice incorporation of halide ions. Energy-dispersive X-ray spectroscopy (EDS) was used as a supportive method to qualitatively confirm the presence and homogeneous distribution of Bi, O, Cl and Br elements in the synthesized samples as depicted in Fig. S1. However, due to the inherent limitations of EDS in accurately quantifying light elements and distinguishing halogens with similar detection sensitivities, compositional determination was not based on EDS quantification. Instead, stoichiometric control combined with XRD phase evolution provides the primary evidence for successful compositional tuning, while EDS serves as complementary confirmation of elemental incorporation and spatial uniformity.

SEM observations were complementary to the efficiency of the acetate-mediated synthesis control in the BiOCl_1−*x*_Br_*x*_ system. As revealed in Fig. 4, BiOCl_1−*x*_Br_*x*_ (*x* = 0.0) first appears as thin, irregular nanosheets. The nanosheets are the result of anisotropic crystalline growth of the layered matlockite structure, which shows lateral ab-plane growth preference. As Cl^−^ was progressively replaced by Br^−^ (*x* = 0.3–0.5), the nanosheets rearranged into a plate-like morphology. The regulated nucleation density and anisotropic crystal development may be a reason for this morphological evolution.

**Fig. 4:**
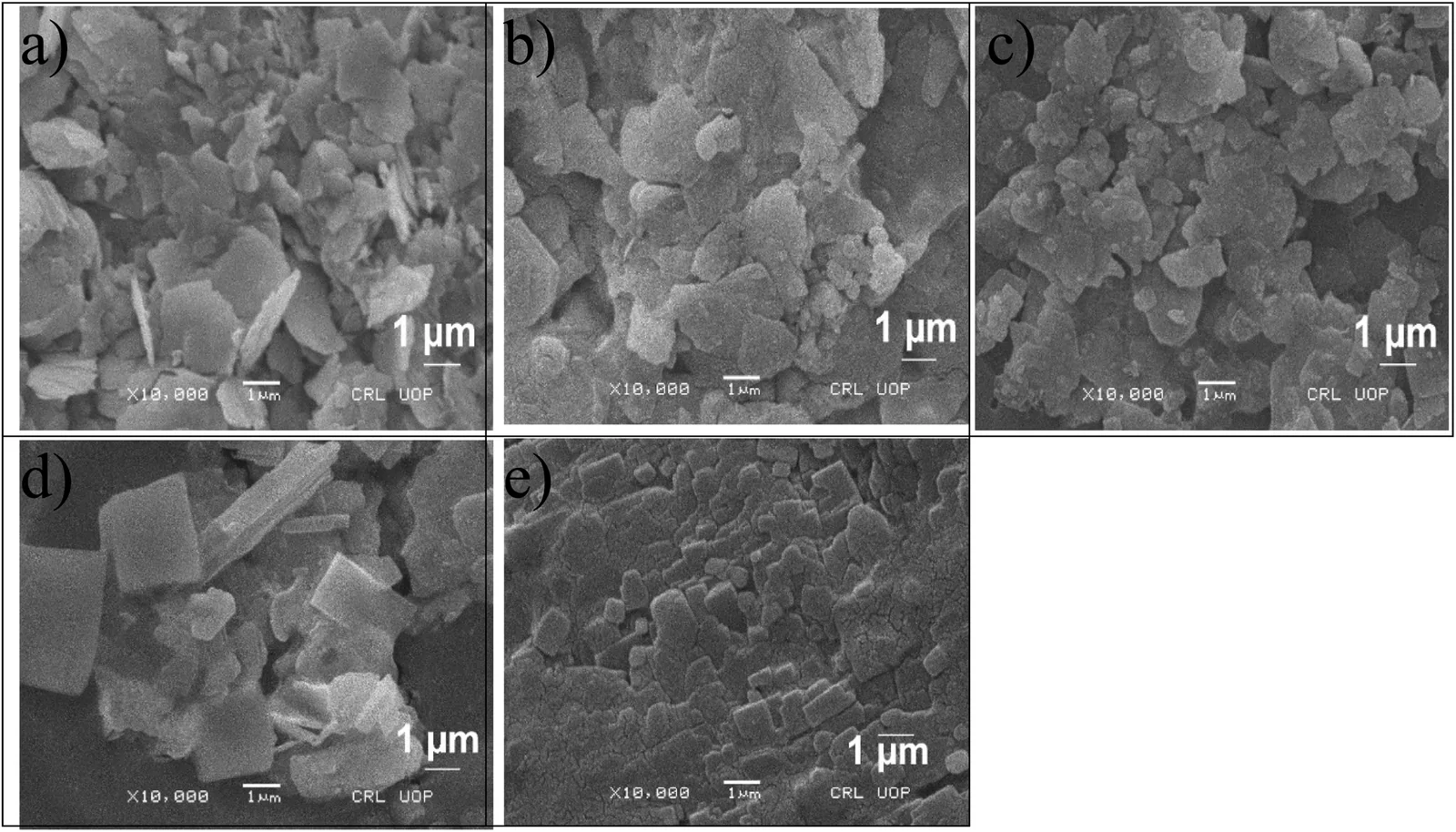
SEM images of BiOCl_1−*x*_Br_*x*_ samples with compositions (a)–(e): *x* = 0.0, 0.3, 0.5, 0.7, 1.0.

As the concentration of Br^−^ was raised, the thickening and aggregation of microsheets were also enhanced. It is consistent with Ostwald ripening, whereby small crystallites dissolve and recrystallize on the larger crystals, thus causing the crystals to grow in size and the particle size to increase. It is interesting to note that under uncontrollable conditions of hydrolysis, the formation of agglomerates is usually irregular, opposite to the current case in the research. The results show that glacial acetic acid is effective as a regulator of hydrolysis, halide incorporation, and crystal growth. The findings confirm that glacial acetic acid is a good controller of hydrolysis, incorporation, and crystal growth. The increase in Br^−^ content (*x* = 0.7–1.0) facilitated the stacking of platelike nanosheets that allow the formation of compact microplates and aggregated microsheets, as previously reported morphologies of BiOX.^30^

A gradual morphological transition between thin nanosheets and thicker assemblies is seen as the Br^−^ content increased in BiOCl_1−*x*_Br_*x*_ (*x* = 1.0). These crystalline microplates in the Br-enriched sample show that nucleation and anisotropic crystal growth are controlled by acetate-assisted hydrolysis. This growth produces less surface roughness and a compact, smooth-surfaced structure. The observation can be explained by a decrease in the amount of surface energy in the process of self-assembly. ImageJ-based particle size analysis (Fig. S2) showed that the average particle size gradually increased from approximately 300 ± 20 nm to 392 ± 20 nm with increasing Br content in BiOCl_1−*x*_Br_*x*_ (*x* = 0.0–1.0).

The composition-dependent morphological evolution mentioned in this research is in contrast with the irregular agglomerates normally generated in uncontrolled hydrolysis. This highlights the importance of acetate in regulating crystal growth of the BiOCl_1−*x*_Br_*x*_ system. Fig. 5 shows that BiOCl_1−*x*_Br_*x*_ powders exhibited redshift in their UV-visible absorption spectra with increasing Br^−^ content (*x* = 0.0 to 0.7). This progressive shift results from modulation of the band structure by halide substitution. Substitution of Cl 3p orbitals with higher-energy Br 4p orbitals is proposed to elevate the valence band maximum and reduce the optical band gap.

**Fig. 5:**
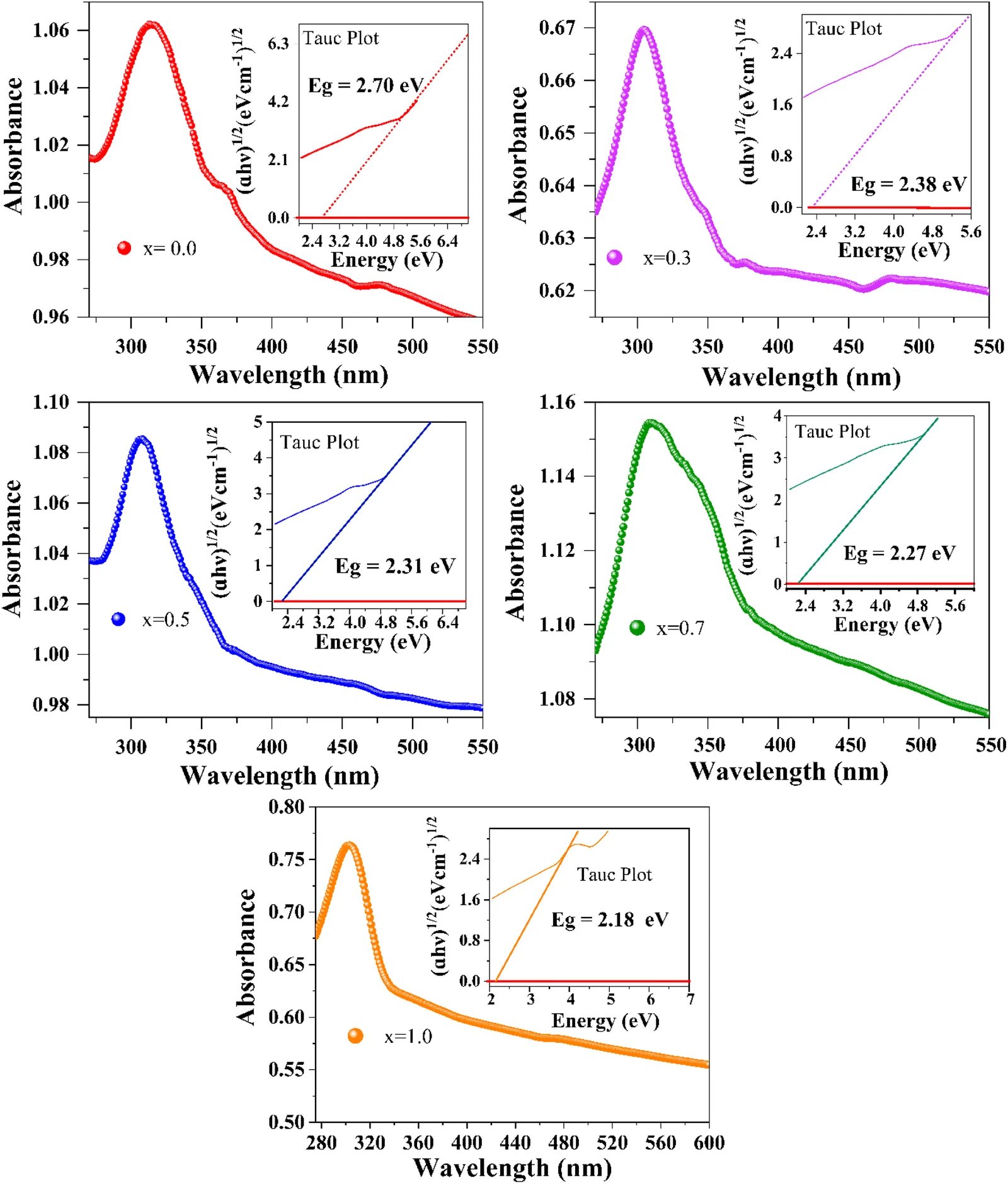
UV visible absorbance spectra and corresponding plots of BiOCl_1−*x*_Br_*x*_ samples (*x* = 0.0, 0.3, 0.5, 0.7, 1.0).

The optical band gap (*E*_g_) was calculated using Tauc's equation:^31^

8 (*αhν*)^*n*^ = *A*(*hν* − *E*_g_)

where *hν* is the photon energy, *α* is the absorption coefficient, A proportionality constant, and *n* corresponds to the nature of the electronic transition. In the present analysis, “*n* = 1/2” was used, corresponding to an allowed indirect transition within the adopted Tauc model. The absorption coefficient was calculated from the measured absorbance using “*α* = 2.303 A/t”, where “*A*” is the absorbance and “*t*” is the optical path length. The optical band gap was determined by extrapolating the linear region of the (*αhν*)^*n*^*versus hν* plots.^32^ The calculated band gaps were 2.70 eV (*x* = 0.0), 2.38 eV (*x* = 0.3), 2.31 eV (*x* = 0.5), 2.27 eV (*x* = 0.7), and 2.18 eV (*x* = 1.0) as shown in Fig. 5, consistent with literature reports showing a similar trend of composition-dependent band gap tuning in BiOCl_1−*x*_Br_*x*_.^10^

The absorption edge arises from intrinsic band-to-band electronic transitions between the O 2p/X np valence and the Bi 6p conduction bands.^17^ A systematic narrowing of the band gap with increasing concentration of Br^−^ indicates a smooth redshift. This shift is attributed to changes in the electronic structure induced by halide substitution.^33^ The bandgap variation in BiOCl_1−*x*_Br_*x*_ originates from two distinct but concurrent contributions. The structural contribution arises from lattice expansion due to substitution of smaller Cl^−^ ions by larger Br^−^ ions, which increases Bi–X bond distances and slightly reduces orbital overlap, leading to, a minor influence on band edge positions. On the other hand, in the dominant contribution, replacement of Cl^−^ (3p) by Br^−^ (4p) alters the valence band structure by enhancing the p-orbital hybridization with the Bi 6s states, leading to a gradual up-shift of the valence band maximum. The conduction band, mostly Bi 6p states, is relatively constant throughout the series. Therefore, the observed bandgap narrowing is governed mainly by halide-induced electronic structure modulation, with lattice expansion playing a secondary role. In addition, compositional changes within the BiOX lattice can be identified by changes in the shape of the peaks. For samples where *x* is 0.0–0.5, the weak shoulder at the absorption edge is believed to be due to the presence of both Cl-rich and Br-substituted [Bi_2_O_2_]-X layers in the same crystal structure. The gradual replacement of the halogen layers creates slightly different environments for Bi–Cl and Bi–Br bonds, resulting in discrete but closely spaced electronic transitions.^15^ These transitions overlap to produce shoulder-like features rather than sudden absorption onsets. This reflects an electronically modulated composition. At *x* = 0.7, the absorption edge is broader due to a more distributed Bi–X bond length. Larger interlayer spacings form as a result of increased Br^−^ incorporation. This *c*-axis growth leads to further development of the bands and increased absorption. The decrease in band gap at *x* = 1.0 suggests that the optical behavior is controlled by the compositional tuning and remains structurally ordered. The absence of sharp absorption edges indicates the fact that compositional-dependent BiOCl_1−*x*_Br_*x*_ materials were formed.

The photoluminescence (PL) emission of BiOCl_1−*x*_Br_*x*_ powders is shown in Fig. 6a. The photoluminescence emission in BiOCl_1−*x*_Br_*x*_ arises from the coexistence of intrinsic band-edge recombination and defect-controlled radiative transitions, whose relative contributions vary with composition. The strong emission in the high-energy UV range of ∼330 nm is attributed to a high-energy radiative transition, but the exact origin of this emission cannot be determined from the present optical and PL measurements. In contrast, the blue emission (∼370–420 nm) may be associated with radiative recombination involving shallow defect states, which may associated with minor lattice imperfections and halogen vacancies within the [Bi_2_O_2_]^2+^ layers. The green to yellow emission (∼420–480 nm) is tentatively linked with defect related localized states that may be formed with the increasing Br content, and they may be related to halogen-vacancy-related states.^34^ The emission at ∼420 nm may be attributed to radiative recombination involving deeper localized defect states, where electron transitions may occur *via* sub-band-gap levels introduced by structural disorder within the BiOCl_1−*x*_Br_*x*_ lattice. These defect states may be correlated with halogen vacancies and changes in the Bi–X bonding environment in the layered [Bi_2_O_2_]^2+^–X^−^. These deeper states may act as trap centres as they lie much lower in the energy band gap than those of shallow defect emissions, allowing the recombination of low-energy radiative electrons. The wide band of emissions at ∼470–483 nm can be explained by the incorporation of band-tail states and high localised defect levels, resulting from the lattice distortion and compositional disorder caused by the Br substitution.^35^ The incorporation of Br^−^ ions can cause more structural perturbation because of ionic size mismatch and the change in the nature of Bi–Br bonding, which may result in an increase in the density of localized states and wider defect distribution. Consequently, the observed visible emission range may reflect a combination of deep defect-related and band-tail-assisted recombination processes rather than a single isolated defect transition. The progressive change in emission color from blue to green-yellow is therefore governed by an increasing contribution of defect-assisted recombination relative to composition-induced modulation of defect density and electronic structure in the BiOX lattice It is seen in Fig. 6b that the PL intensity changes with Br^−^ content, and the highest PL intensity is obtained for *x* = 1.0, which shows strong composition dependence of the radiative emission behaviour. The results show that the emission wavelength can be tuned in these materials with composition changes and suggest that BiOCl_1−*x*_Br_*x*_ could be used in luminescent applications.

**Fig. 6:**
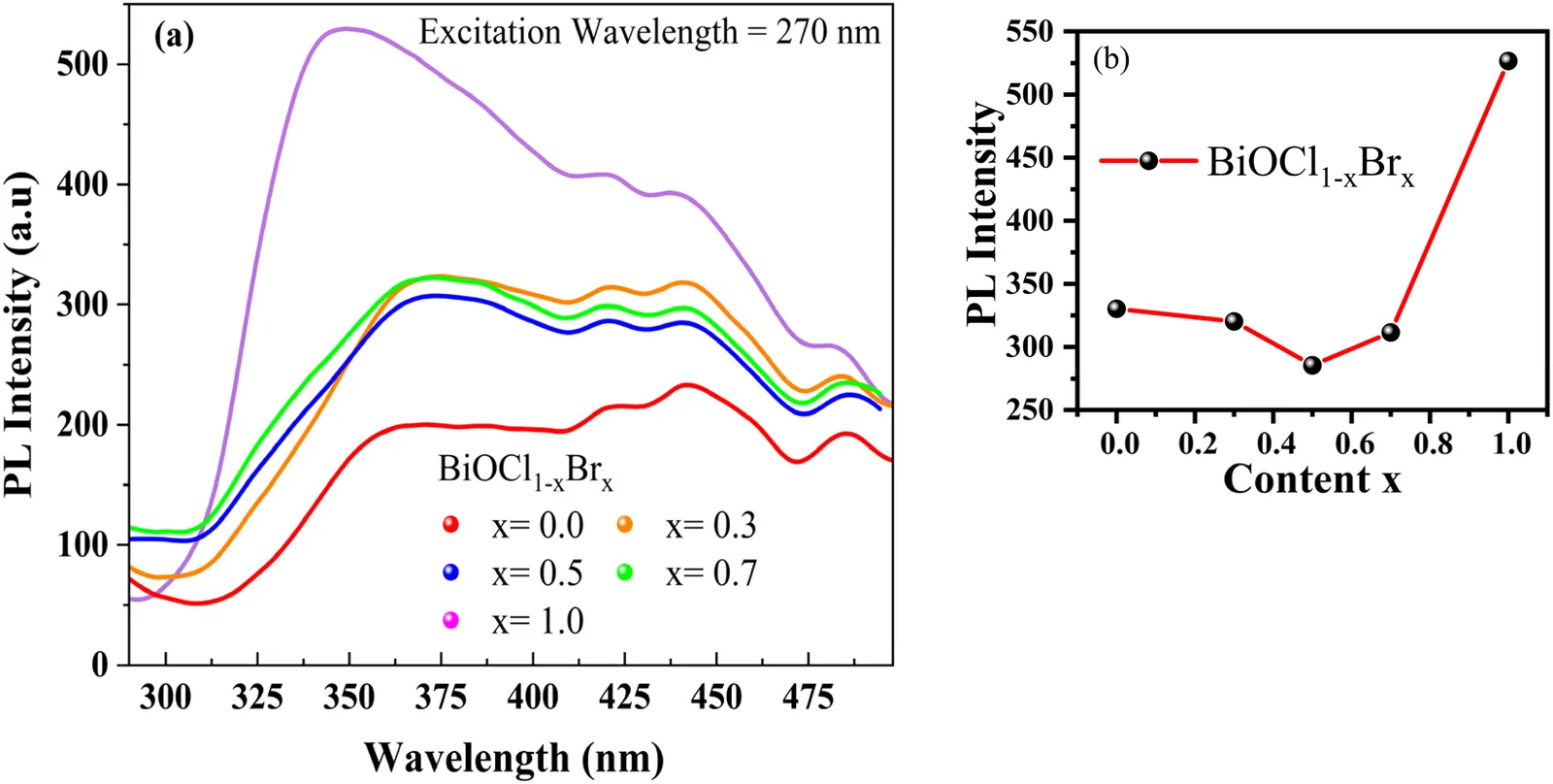
(a) PL emission spectra of BiOCl_1−*x*_Br_*x*_ under 270 nm excitation; (b) PL intensity variation with Br^−^ content.

Fig. 7 suggests a likely luminescence mechanism of BiOCl_1−*x*_Br_*x*,_ in which the proposed scheme is applied to determine the correlation between the development of the UV-visible band gap and the emitted PL. To analyse the observed emission bands further, the energies of PL emission were calculated using the formula “*E*_PL_ = 1240/*λ*. The energies obtained suggest that the measured emissions are not directly attributable to the defect level positions by the available optical data. So, the suggested defect-related transitions are discussed qualitatively, and the exact energetic positions and formation energies must be calculated using first principles.

**Fig. 7:**
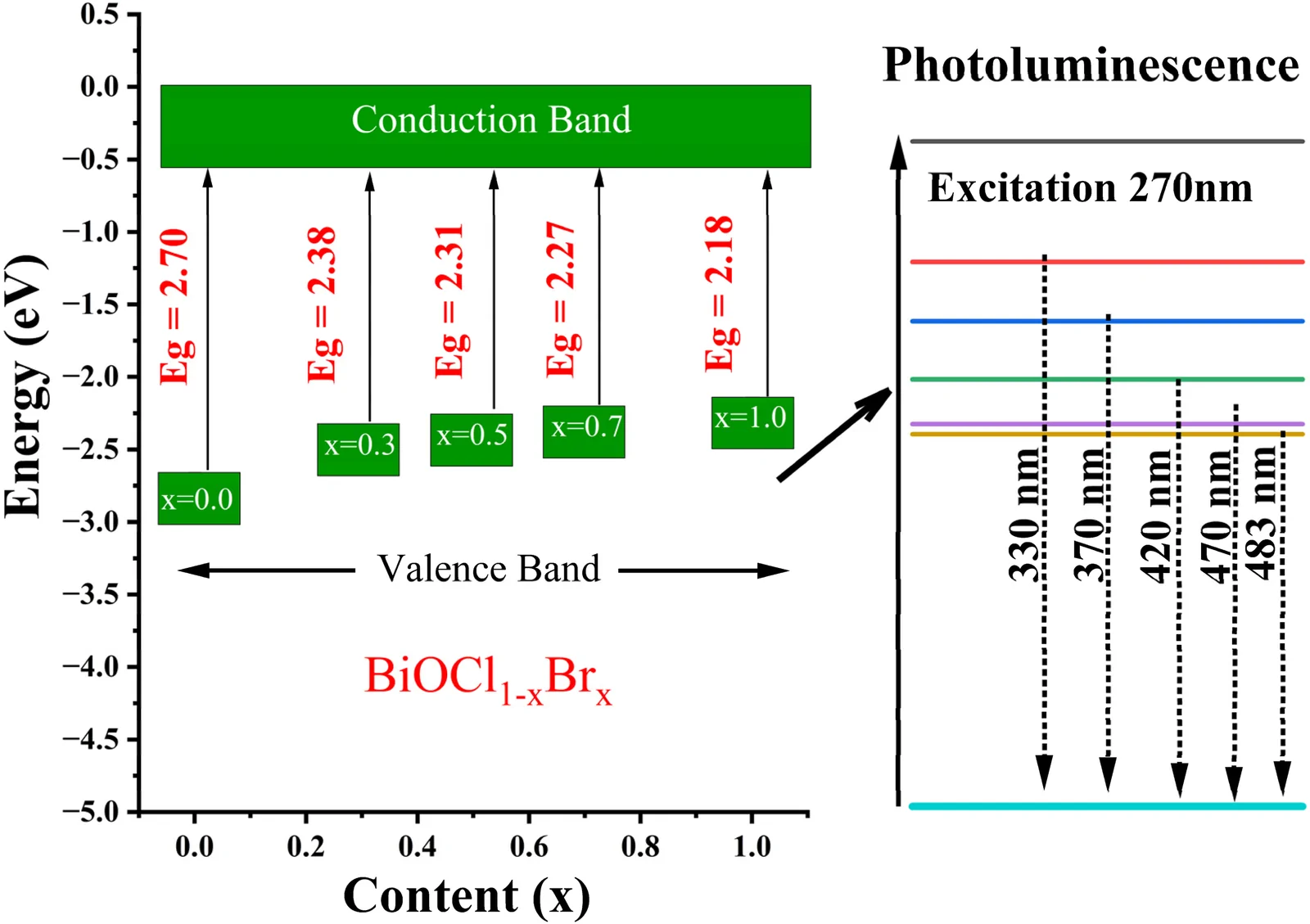
Schematic illustration of proposed luminescence mechanism in BiOCl_1−*x*_Br_x_.

### 3.1. CIE chromaticity analysis

The Commission Internationale de l’Eclairage (CIE) chromaticity coordinates of BiOCl_1−*x*_Br_*x*_ (*x* = 0.0–1.0) phosphors are calculated from the PL emission spectra under 270 nm excitation as shown in Fig. 8. The chromaticity coordinates exhibit a systematic shift with increasing Br^−^ content, indicating a gradual emission color transition from blue to green-yellow, and finally to green.

**Fig. 8:**
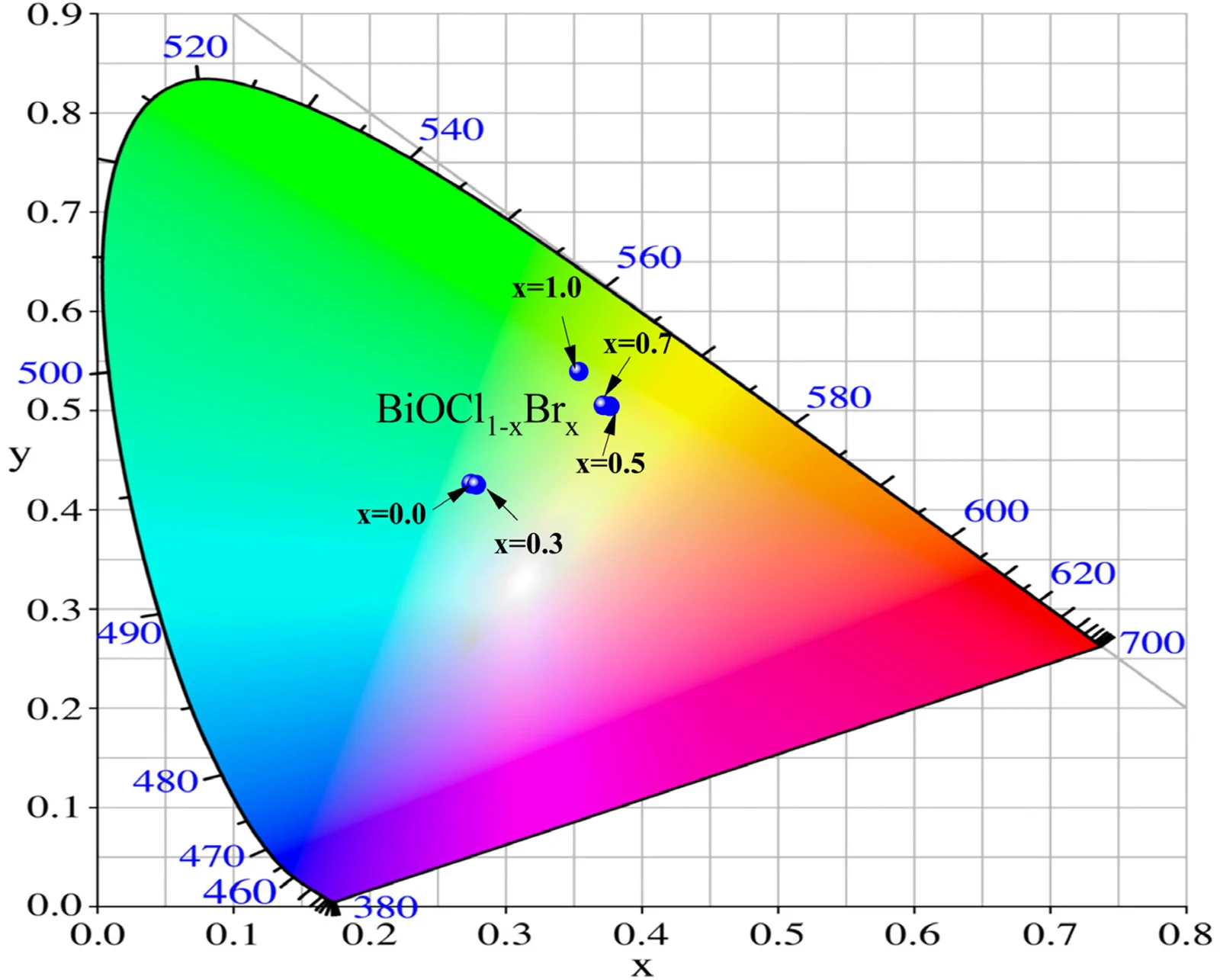
CIE chromaticity diagram of BiOCl_1−*x*_Br_*x*_ (*x* = 0.0–1.0).

This compositional color tuning can be related to the band structure modulation and distribution of defect-states inferred from UV-vis absorption and PL emission results. As revealed in the UV spectra, substitution of Cl^−^ with Br^−^ modifies the valence band by increasing the Br^−^ 4p orbital contribution. This leads to modification in the electronic band structure and the formation of localized defect states within the band gap. These defect states act as radiative recombination centers, responsible for broadband visible emissions.

In Cl-rich compositions (*x* = 0.0–0.3), near band-edge transitions and shallow defect transitions (∼330–370 nm) dominate the emission, producing a more significant contribution at shorter wavelengths and bluer emissions. In the intermediate compositions (*x* = 0.5–0.7), increased lattice distortion and defect density enhance the contribution of deep trap-assisted emissions (∼420–480 nm), shifting the emission spectrum toward longer wavelengths and moving the chromaticity coordinates toward the green-yellow region.^36^

For the Br-rich composition (*x* = 1.0), the emission is dominated by defect-assisted visible transitions primarily centered in the green spectral region. Although Br incorporation increases structural relaxation, it may reduces excessive deep-trap disorder, leading to a more stabilized defect distribution and a narrowed emission envelope, which prevents further redshift toward yellow. As a result, chromaticity coordinates are positioned in the green region rather than shifting further toward yellow. The uncertainty in the CIE coordinate is estimated as ± 0.002 in both the *x* and *y* directions, as shown in Table 3.

**Table 3:** CIE chromaticity coordinates, BiOCl_1−*x*_Br_*x*_ color purity, and CCT (*x* = 0.0, 0.3, 0.5, 0.7, 1.0)

NO.	BiOCl_1−*x*_Br_*x*_	CIE (*x*, *y*) ± 0.002	CCT (*K*)	Color purity % ± 5%
1	*x* = 0.0	0.301, 0.307	7528 ± 250	82%
2	*x* = 0.3	0.305, 0.306	7244 ± 200	81%
3	*x* = 0.5	0.403, 0.385	3507 ± 70	83%
4	*x* = 0.7	0.398, 0.386	3610 ± 100	83%
5	*x* = 1.0	0.380, 0.420	4293 ± 100	83%

The correlated color temperature (CCT) of the samples is determined using the following empirical relation:^37^

9 CCT = −437n^3^ + 360 1n^2^ − 686 1n + 5514.31

where10
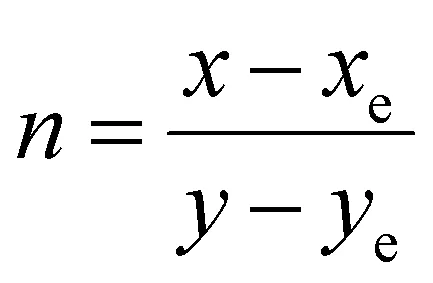
And (*x*, *y*) are the color coordinates of the obtained phosphors, while (*x*_e_, *y*_e_) = (0.3320, 0.1858) represents the standard CIE illuminant. The CCT error is estimated based on the ± 0.002 uncertainty in the CIE coordinates (*x*, *y*) and the spectral width of PL emissions.

Furthermore, the color purity (CP) is calculated using equation.:^38^11
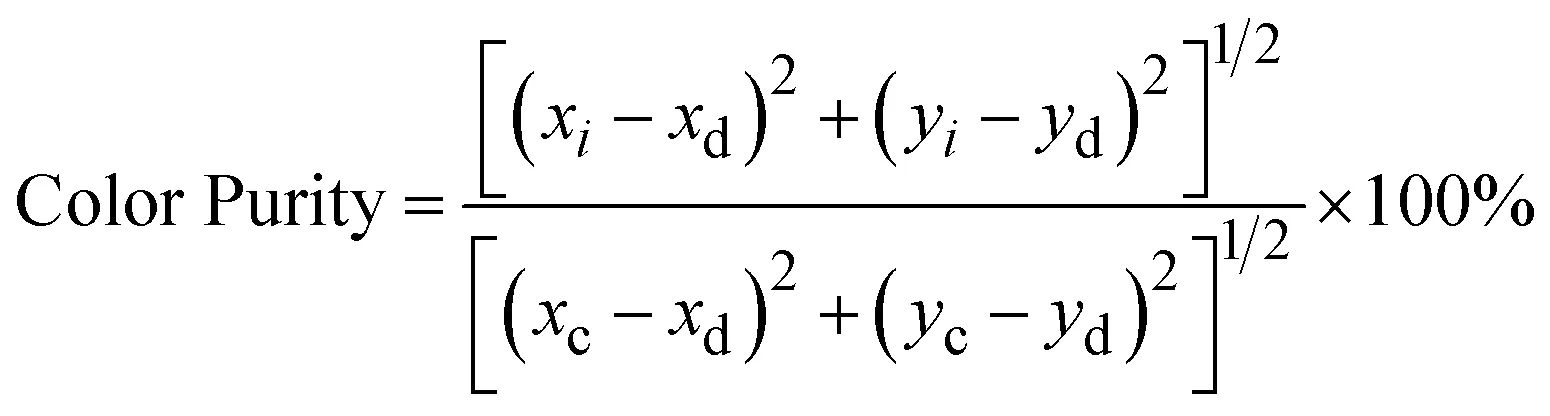
where (*x*_i_, *y*_i_) denote the chromaticity coordinates of the emission light, (*x*_d_, *y*_d_) = (0.316, 0.316) represent the coordinates of the CIE standard white illuminant, and (*x*_c_, *y*_c_) correspond to the dominant wavelength. The estimated error in color purity calculated from the spectral width of the PL emissions is ±5%.

The calculated CCT and color purity values indicate that the emission color of BiOCl_1−*x*_Br_*x*_ can be effectively tuned through halide substitution. These results demonstrate the potential of BiOCl_1−*x*_Br_*x*_ materials for emission color modulation. The color purity values above 80% indicate relatively saturated emission colors, supports the effectiveness of halide composition in tuning the emission color, as summarized in Table 3. This tunable emission behavior, along with high color purity, demonstrates the potential of BiOCl_1−*x*_Br_*x*_ solids for multicolor luminescent phosphor and optoelectronic applications.

## Conclusion

The work demonstrated that the halide composition is a key parameter for controlling the emission color in mixed-halide BiOCl_1−*x*_Br_*x*_ materials. Acetic acid-assisted controlled hydrolysis enabled the formation of mixed halides across the investigated composition spectrum (*x* = 0.0–1.0). The lattice expansion, band modulation, and defect-assisted photoluminescence resulted in emission color tuning from blue to green-yellow. The materials exhibited color purity exceeding 80 ± 5%, indicating relatively saturated emission colors. Unlike strategies based on multiple dopants or heterostructures, this study demonstrated that intrinsic halide chemistry, regulated *via* acetate-controlled hydrolysis, plays a key role in achieving tuned emissions. Thus, a clear structure–property correlation is maintained, which allows tunable emission colors. The results establish mixed-halide BiOCl_1−*x*_Br_*x*_ as a general composition-dependent luminescent material.

The present study further demonstrates that systematic halide substitution provides a direct pathway for tailoring lattice structure, defect-state distribution, electronic band structure, and visible-light emission within BiOX framework. This acetate-assisted hydrolysis route is simple and effective and can be used to facilitate defects and emission tunability without the need for external dopants, surfactants or complex heterostructures. These results emphasize the general prospect for the use of mixed-halide BiOCl_1−*x*_Br_*x*_ materials in various tunable phosphors, photonic and optoelectronic applications, as well as uncovering an essential structure–property relationship in BiOX materials with composition-dependent luminescence.

While defect-assisted luminescence was interpreted from the optical analysis, more sophisticated techniques like XPS, EPR are needed to confirm the presence of defect states and recombination pathways within BiOCl_1−*x*_Br_*x*_.

## Conflicts of interest

There is no conflict to declare.

## Supplementary Material

NA-OLF-D6NA00183A-s001

## Data Availability

All data supporting this article have been provided within the main content of this manuscript. No additional data are available outside of this publication. Supplementary information (SI) is available. See DOI: https://doi.org/10.1039/d6na00183a.
