# Supplementary material for: Acetic acid-assisted controlled hydrolysis for Br-content-dependent emission color tuning in mixed-halide BiOCl1−xBrx systems
Source: Nanoscale Adv. 2026 Oct 27. Online ahead of print. doi: 10.1039/d6na00183a (PMC13712237; doi:10.1039/d6na00183a)
Supplement: Supplementary file 1 [file NA-OLF-D6NA00183A-s001.pdf]

Supplementary Information (SI) for Nanoscale Advances.
This journal is © The Royal Society of Chemistry

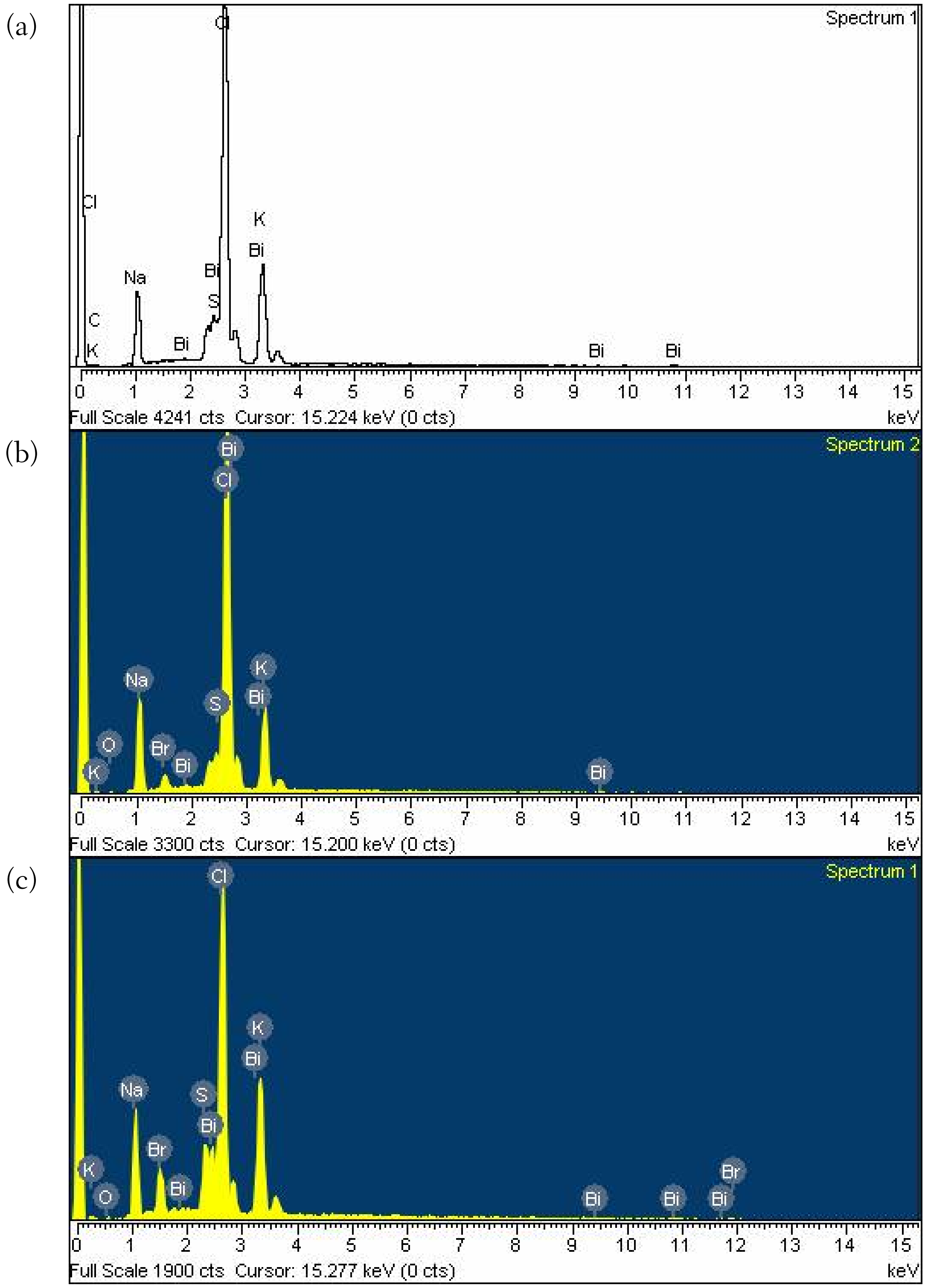

(d)

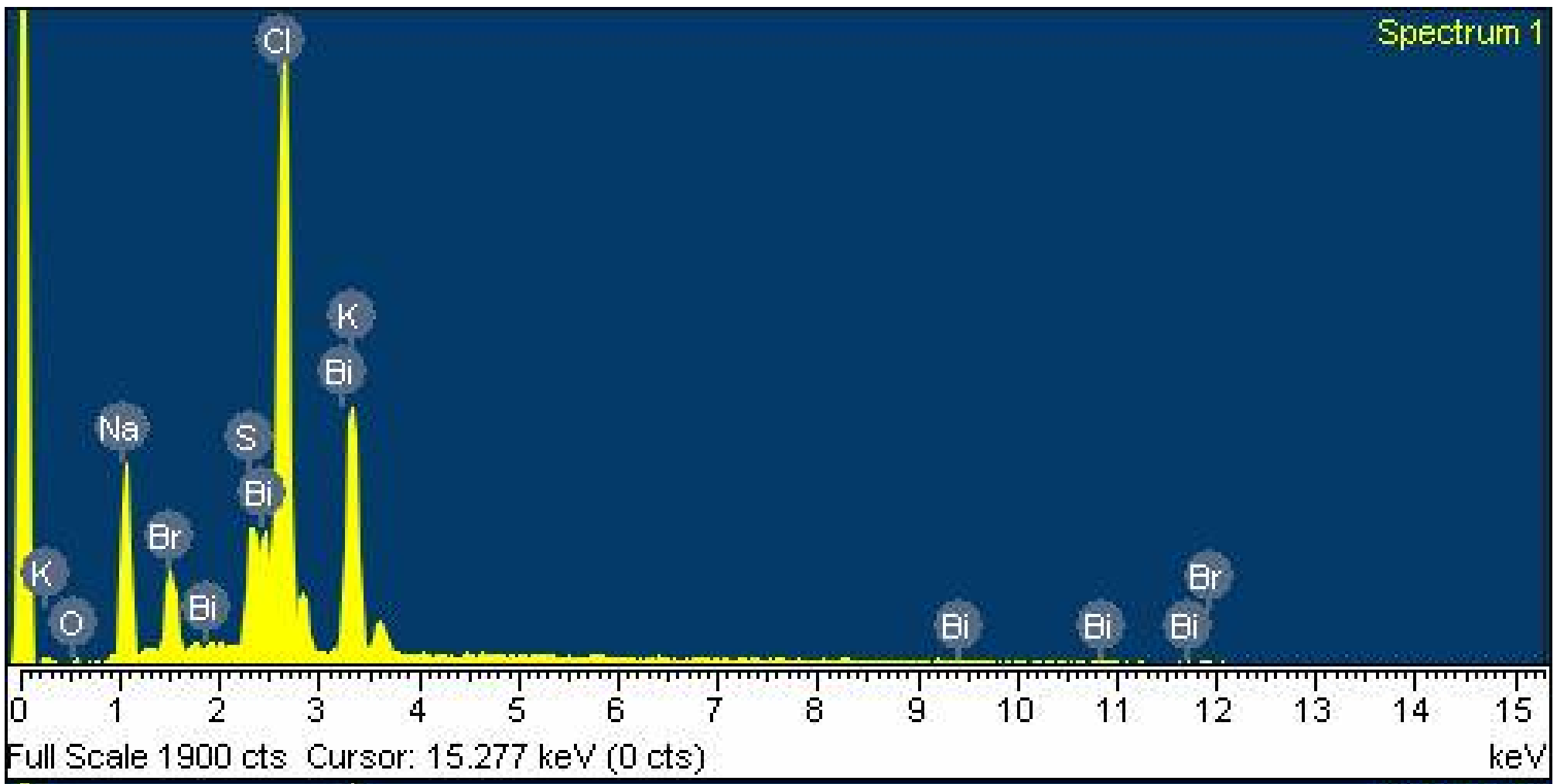

(e)

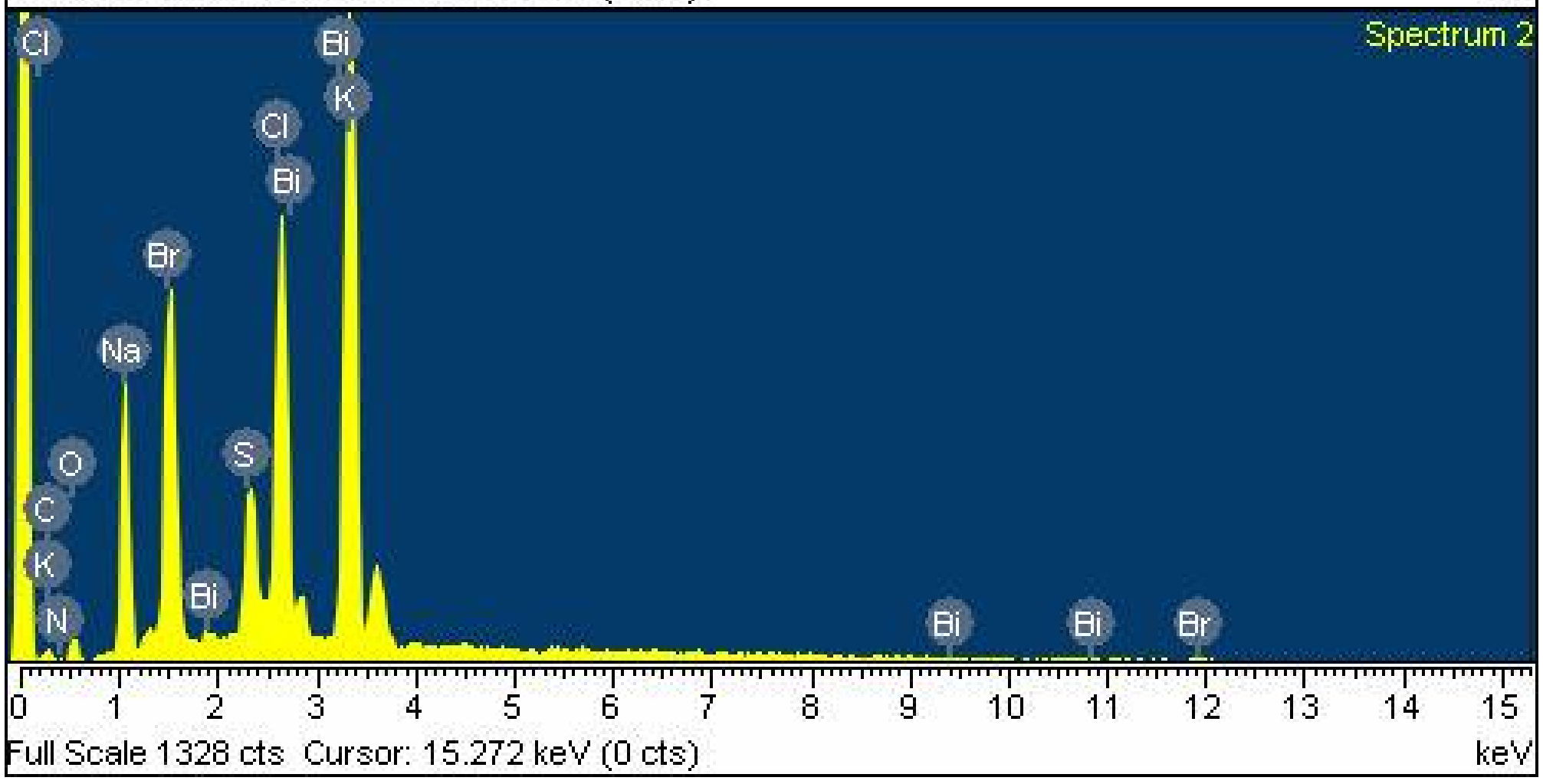

Figure S1: EDS images of $BiOCl_{1-x}Br_x$ samples with compositions (a)–(e): x=0.0, 0.3, 0.5, 0.7, 1.0.

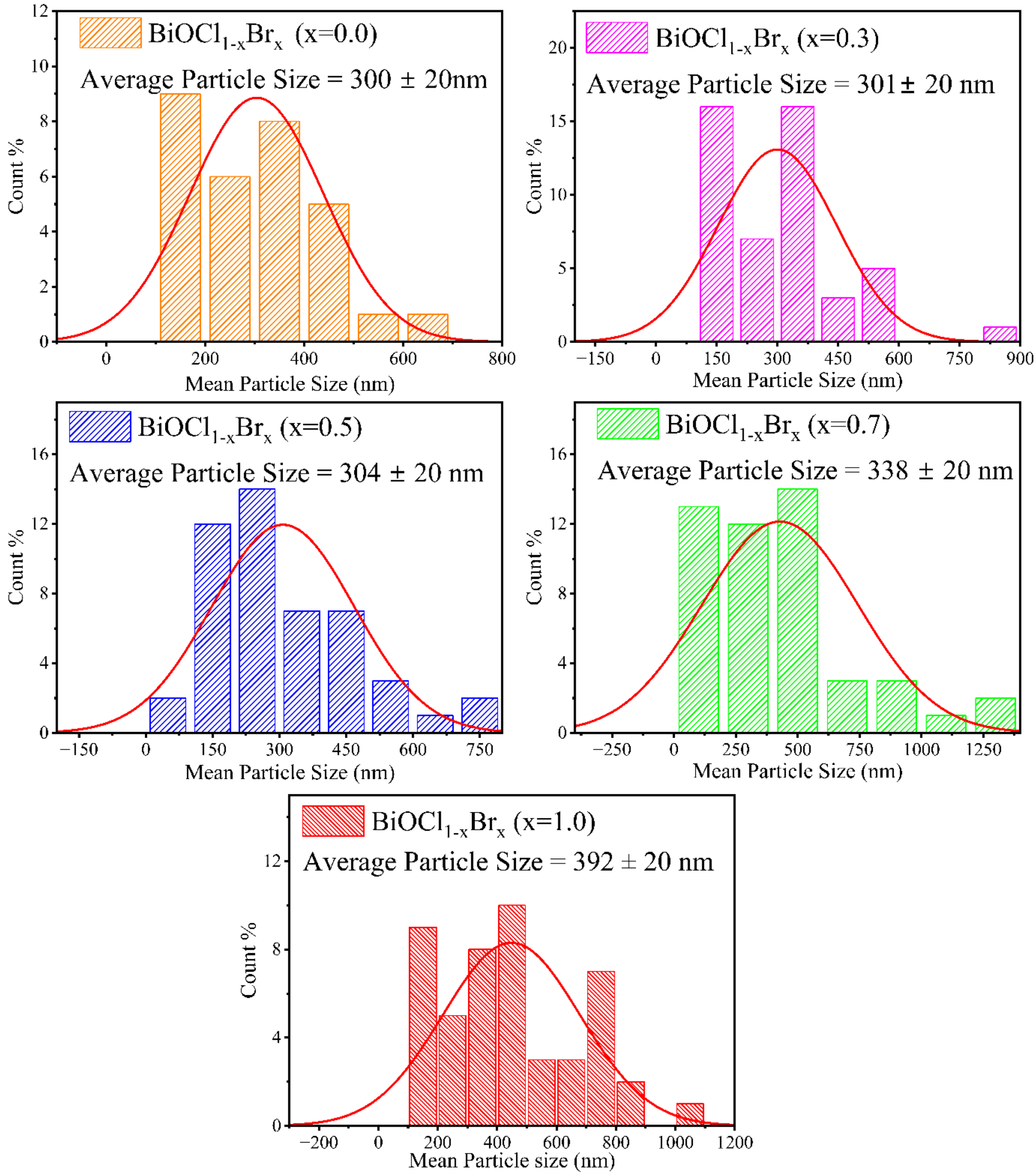

Figure S2: ImageJ-based particle size distribution histograms of $BiOCl_{1-x}Br_x$ (x = 0.0–1.0) powders obtained from SEM micrographs (a)–(e): x=0.0, 0.3, 0.5, 0.7, 1.0.
